# Primary Amenorrhea With Hypothyroidism: Finding the Cause

**DOI:** 10.7759/cureus.20285

**Published:** 2021-12-08

**Authors:** Asim Munir Alvi, Ahmed Imran Siddiqi, Umal Azmat, Waqas Shafiq, Sardar A Khan

**Affiliations:** 1 Endocrinology, Diabetes and Metabolism, Shaukat Khanum Memorial Cancer Hospital and Research Centre, Lahore, PAK; 2 Endocrinology and Diabetes, Shaukat Khanum Memorial Cancer Hospital and Research Centre, Lahore, PAK; 3 Endocrinology, Shaukat Khanum Memorial Cancer Hospital and Research Centre, Lahore, PAK

**Keywords:** turner syndrome, hypergonadotropic hypogonadism, hypothyroidism, multinodular goiter, primary amenorrhea

## Abstract

Primary amenorrhea is a serious medical condition. A thorough clinical assessment is necessary for a timely and correct diagnosis and management of this ailment to prevent long-term health and social problems. Turner's syndrome is considered one of the important causes of primary amenorrhea, with an incidence of one in 2,500 to one in 3,000 live-born girls. In this report, we present an interesting case involving multinodular goiter, hypothyroidism, and primary amenorrhea.

A 24-year-old woman with a history of multinodular goiter and hypothyroidism attended an endocrine clinic with fine-needle aspiration cytology (FNAC) report of her bilateral thyroid nodules, which showed Bethesda category IV. She had a history of learning difficulties. During detailed history-taking, the patient also complained of primary amenorrhea. Clinical examination showed a lack of secondary sexual characters. Biochemical, imaging, and cytogenetic investigations were suggestive of absent ovaries and fallopian tubes, streaked uterus, hypergonadotropic hypogonadism, and X0 karyotyping.

The learning objectives of this case report are as follows: firstly, in countries with a lack of awareness and limited health resources, patients may present with one of the manifestations of Turner's syndrome. Clinicians from all specialties should be aware of the clinical features of this relatively rare entity and should try to make the most of incidental clinical findings. Secondly, clinicians should be more vigilant and thorough in their clinical assessment of patients with learning difficulties to minimize the chances of missing a clinical diagnosis.

## Introduction

Amenorrhea is a serious clinical problem both for patients and clinicians and is defined as the absence of menstruation in females of reproductive age [1]. Primary amenorrhea is characterized by the absence of menarche (start of menstruation) in females of 15 years or older having secondary sexual characters and normal growth or the absence of menarche in females of 13 years or older without signs of pubertal development [2]. Secondary amenorrhea is defined as the loss of regular menses for three months or loss of irregular menses for six months after menarche [3].

According to the World Health Organization (WHO), amenorrhea is the sixth most common cause of infertility, and around 2-5% of adolescent girls are affected by primary amenorrhea. Its incidence has been on the rise due to an increase in general awareness, improving health facilities, and decreasing trend of child marriages [1]. Primary amenorrhea can occur due to pituitary disorders resulting in hypogonadotropic hypogonadism, disorders of gonads leading to hypergonadotropic hypogonadism, other endocrine glands diseases, or uterovaginal anomalies. Clinical investigations of primary amenorrhea are warranted when there is no menstruation for three years after thelarche (start of breast development) or after five years of thelarche if it has happened before the age of 10 years [2]. We discuss an interesting case of a patient who presented with multinodular goiter, hypothyroidism, and primary amenorrhea.

## Case presentation

A 24-year-old woman, diagnosed with multinodular goiter and hypothyroidism, presented to the endocrine clinic. She was already on thyroxin replacement. She was also clinically and biochemically euthyroid. Her fine needle aspiration cytology (FNAC) report of bilateral thyroid nodules showed Bethesda category IV. Primary amenorrhea was an incidental finding in her case. There was no evidence of stress, anxiety, depression, or weight changes. She had no history of strenuous exercise or abnormal eating behavior. She denied any history of cyclical abdominal pain, head trauma, headache, visual problems, and galactorrhea. There was no history of radiation to the brain and chemotherapy. She had been born of consanguineous marriage. All her nine sisters had achieved menarche between the ages of 12 and 13 years. Her mother also had normal menstrual history. The patient had learning difficulties and had not attended school due to this.

On examination, she had short stature with a webbed neck. She had minimal axillary hair, pubic hair (Tanner stage 1), no breast development (Tanner stage 1), a high arch palate, and squint. The external genital examination was unremarkable. Her BMI was 20 kg/m^2^. Table 1 illustrates her hormonal profile, as well as imaging and karyotyping results.

**Table 1:** Hormonal profile, imaging, and karyotyping

Test name	Value	Reference range
Luteinizing hormone (LH)	17.6 mIU/mL	1.1–14.7
Follicle-stimulating hormone (FSH)	130 mIU/mL	2.8–11.3
Estradiol	28.67 pg/mL	19.5–214
Dehydroepiandrosterone sulfate (DHEA-SO4)	68.9 ug/dL	35–430
Prolactin	11.4 ng/mL	1.90–25
Testosterone	14.28 ng/dL	12–59.4
Cortisol	17.52 mcg/dL	5.27–22.45
Thyroid-stimulating hormone (TSH)	5.35 uIU/mL	0.35–5.5
Free T4	1.01 ng/dL	0.89–1.76
Ultrasound pelvis	Absent cervix, uterus, and bilateral ovaries	
Magnetic resonance imaging (MRI) pelvis	Extremely rudimentary uterus with streak-like configuration. No ovarian tissue was identified	
Echocardiography	Unremarkable study	
Ultrasound thyroid	Multinodular goiter	
Karyotyping	X0	

**Figure 1:**
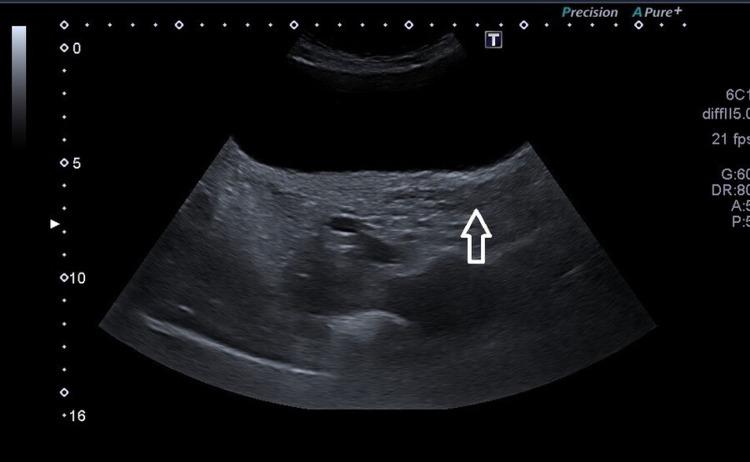
Ultrasound pelvis (arrow showing rudimentary uterus)

**Figure 2:**
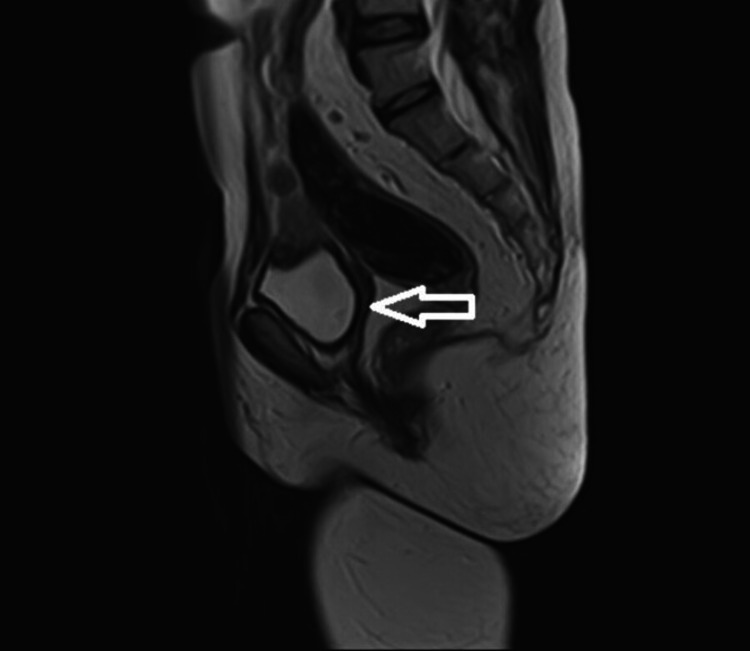
MRI abdomen and pelvis (arrow showing rudimentary uterus) MRI: magnetic resonance imaging

Biochemical investigations confirmed hypergonadotropic hypogonadism, and imaging investigations (Figures 1, 2) confirmed absent ovaries, fallopian tubes, and streaked uterus.

**Figure 3:**
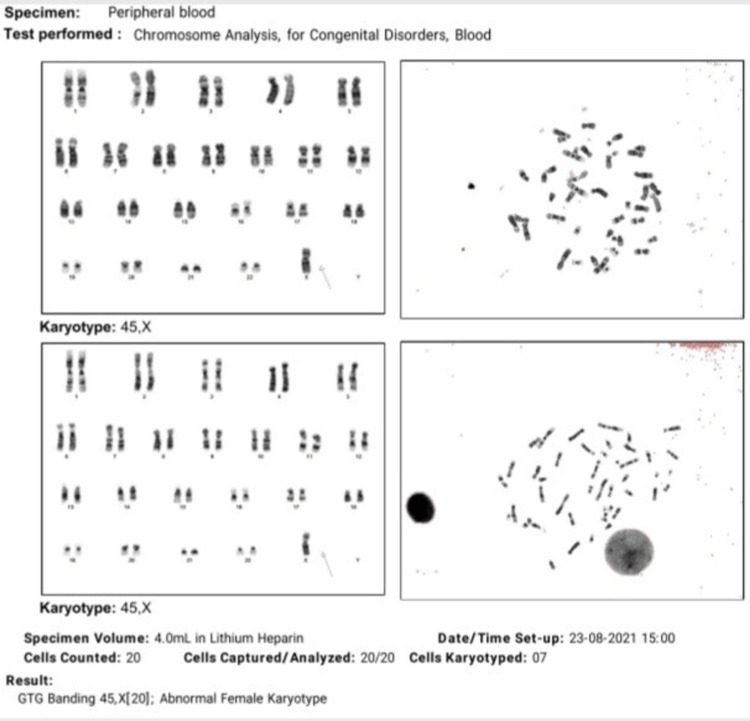
Karyotyping (showing X0 karyotype)

X0 karyotyping result confirmed our clinical suspicion of Turner’s syndrome (Figure 3).

The patient is currently receiving psychological support and counseling regarding her diagnosis and subsequent emotional issues with the support of her family. She was prescribed hormone replacement therapy with ethinylestradiol/levonorgestrel. We offered total thyroidectomy for her multinodular goiter in light of her bilateral Bethesda category IV nodules and she consented. Histopathology of thyroid tissue has shown adenomatous colloid goiter, and she is presently doing well under regular follow-up in the endocrinology department.

## Discussion

There are many causes of primary amenorrhea, including hypogonadotropic hypogonadism, hypergonadotropic hypogonadism, and normogonadotropic hypogonadism (Table 2) [2-4].

**Table 2:** Causes of primary amenorrhea CAH: congenital adrenal hyperplasia; IBD: inflammatory bowel disease; PCOS: polycystic ovarian syndrome; SOD: septo-optic dysplasia; PWS: Prader-Willi syndrome; LMMBS: Laurence-Moon-Bardet-Biedl syndrome; AIS: androgen insensitivity syndrome; OCPs: oral contraceptive pills; SSRIs: selective serotonin reuptake inhibitors

Hypogonadotropic hypogonadism	Hypergonadotropic hypogonadism	Normogonadotropic hypogonadism
Constitutional delay/self-limited delayed puberty	Premature ovarian insufficiency/failure (Turner's syndrome, gonadal dysgenesis, autoimmune oophoritis, polyglandular autoimmune syndrome, radiation therapy, surgery, chemotherapy, infections)	Pituitary (hyperprolactinemia)
Congenital hypogonadotropic hypogonadism (Kallmann syndrome)		Adrenals (non-classic CAH, androgen-secreting tumors, Addison's disease)
Functional hypothalamic problems (anorexia nervosa, chronic diseases like celiac disease, IBD, strenuous exercise, stress, psychiatric issues)		Ovaries (PCOS, androgen- or estrogen-secreting tumors)
Syndromic congenital hypogonadotropic hypogonadism (CHARGE syndrome, CAH, SOD, PWS, LMBBS)		Uterus (Müllerian structures agenesis, cervical agenesis, AIS, pregnancy)
Pituitary hormone deficiencies due to its destruction (benign adenomas and other tumors; cysts, infiltrative disorders, previous surgery, head trauma, pituitary apoplexy, vascular lesions, empty sella syndrome, radiation therapy)		Vagina (atresia, transverse septum, distal atresia)
Thyroid problems (hypothyroidism or hyperthyroidism)		Imperforation of hymen
Medications (anesthetics, anticonvulsants, antipsychotics, opiates, anti-emetics, SSRIs, OCPs, alcohol abuse, heroin, cocaine, steroids, androgens use)		

A systematic clinical approach is key to a proper assessment of patients presenting with primary amenorrhea, including thorough clinical history, complete physical examination, and examination of external and internal genitalia. Lab work should include thyroid-stimulating hormone (TSH), follicle-stimulating hormone (FSH), luteinizing hormone (LH), estradiol, testosterone, beta-human chorionic gonadotropin (B-HCG), serum prolactin levels, and karyotyping (if clinically indicated. Perform karyotype in all patients of hypergonadotropic hypogonadism and with androgenic features) [2]. The hormonal profile of our patient showed hypergonadotropic hypogonadism, and this prompted us to perform karyotyping.

After complete history-taking, physical examination, and initial investigations of our patient, two main differentials were made: Turner's syndrome and Müllerian agenesis with ovarian failure as the cause of primary amenorrhea. In the ultrasonography pelvis report of our patient, ovaries and uterus were absent; Müllerian agenesis with ovarian failure was one of our differentials as there was a case reported in the literature that showed Müllerian agenesis with ovarian failure [5].

Patients with only Müllerian agenesis without ovarian failure (or gonadal dysgenesis) have normal secondary sexual characters with normal LH, FSH, and estradiol levels because gonads have different embryonic origins [5-6]. If secondary sexual characters are absent, together with the absence of internal sexual structure, then the diagnosis will be Müllerian agenesis [Mayer-Rokitansky-Küster-Hauser (MRKH) syndrome with gonadal dysgenesis] [5].

Turner’s syndrome can present with primary amenorrhea and lack of secondary sexual characters if ovarian failure develops before the onset of puberty [7]. The lack of secondary sexual features in our patient was due to the absence of estrogen (ovarian failure) before the onset of puberty. About half of Turner's syndrome patients have only one X chromosome (45 X0) and 5-10% have a doubling of the long arm of one X (46,X,i(Xq)). Most of the other patients have mosaicism for 45, X, with one or more additional cell lineages [7]. Karyotyping result of our patient showed an X0 karyotype, which confirmed the diagnosis of Turner's syndrome. The diagnosis of Turner’s syndrome should be considered in any teenage girl with primary or secondary amenorrhea, especially when she is short-statured. Clinically, Turner’s syndrome can involve any system of the body (general, cardiovascular, endocrine, renal, gastrointestinal, musculoskeletal, dermatological, eye, and otorhinolaryngological) and can present with manifestations related to any system of the body (Table 3) [7].

**Table 3:** Clinical features of Turner's syndrome GERD: gastroesophageal reflux disease; IBD: inflammatory bowel disease

Systems/problems	Features that can be present in Turner’s syndrome
General	Short stature, mostly normal intelligence, female phenotype
Cardiovascular	17-45% have congenital heart diseases; aortic coarctation and bicuspid aortic valve are most common; others include left-sided cardiac defects, hypertension, mitral valve prolapsed, and conduction defects
Endocrine	Hypothyroidism; gonadal dysgenesis is a cardinal feature of Turner's syndrome; infertility, diabetes mellitus
Eye, ENT problems	Strabismus, ptosis, cataract, nystagmus, color blindness, recurrent otitis media, sensorineural hearing loss
Gastrointestinal problems	GERD, IBD, celiac disease, gall bladder disease
Renal problems	Structural renal problems (horseshoe-shaped kidney, duplication of collecting system)
Musculoskeletal problems	Dislocation of patellar bone and pain in the knee joint; deformity of the ulnar head causes the increase-carrying angle of the arm and may result in a decreased range of motion, Madelung's deformity, and congenital dislocation of the hip, high arched palate, spinal deformities, and osteoporosis
Dermatological problems	Congenital puffiness of hands and feet, increased melanocytic nevi, increased risk of keloid formation, premature fine wrinkling of facial skin
Neoplasm	Colon cancer, gonadoblastoma, dysgerminoma, risk of endometrial carcinoma in patients with a history of unopposed estrogen treatment

Of the clinical features associated with Turner's syndrome, our patient had short stature, a webbed neck, hypothyroidism, strabismus, a high arch palate, and excessive melanocytic nevi. After a diagnosis of Turner's syndrome is made, screening for other conditions associated with Turner's syndrome is necessary when clinically indicated.

## Conclusions

Primary amenorrhea can result from various causes, and through broad vision, and proper and thorough clinical assessment, clinicians can timely diagnose and treat the cause of this condition. The reason for the delayed diagnosis in our case was most likely the low IQ of our patient and possibly socioeconomic issues. The learnings from this case report are as follows: primarily, in countries where healthcare-related awareness is limited and health resources are scarce, patients may present with one of the manifestations of Turner's syndrome. Clinicians from all specialties should be mindful of the characteristic clinical features of this uncommon entity and should do their best to make the best use of incidental clinical findings. Secondly, clinicians should employ extreme vigilance and thoroughness in their clinical assessment of patients with learning difficulties so that a clinical diagnosis of primary amenorrhea is not missed.
